# Mondor’s disease as a complication in breast surgery in a male patient. The first ever reported case in literature

**DOI:** 10.1080/23320885.2022.2121711

**Published:** 2022-09-13

**Authors:** Mario Faenza, Tommaso Pelella, Andrea Maria Antonetti, Sara Izzo, Roberto Grella, Giuseppe Andrea Ferraro

**Affiliations:** Plastic Surgery Unit, Multidisciplinary Department of Medical Surgical and Dental Sciences, University of Campania 'Luigi Vanvitelli’, Naples, Italy

**Keywords:** Gynecomastia, complication, thrombophlebitis, body contouring

## Abstract

Mondor’s disease (MD) is an uncommon clinical condition characterized by thrombophlebitis of the superficial veins of the anterolateral thoracoabdominal wall. In this paper we present the first ever reported case of Mondor’s disease in male patient after surgical correction of gynecomastia with liposuction assisted skin sparing adenectomy.

## Introduction

Mondor’s disease (MD) is an uncommon and self-limiting clinical condition characterized by thrombophlebitis of the superficial veins of the anterolateral thoracoabdominal wall causing the sudden onset of chest pain and palpable subcutaneous cords [1,2].

MD has been described in several anatomic regions but chest wall is the most frequent location and is strictly related to breast surgery in female patients[3].

A similar condition has been reported in male patients at penile level (Penile Mondor’s Disease) and is related to vessel damage after trauma or surgical procedures [4,5].

Gynecomastia is defined as benign proliferation of glandular breast tissue in men and often causes emotional distress and physical discomfort [6,7].

Many surgical techniques have been described for treatment of gynecomastia from simple adenectomy to reduction mammaplasty with dermoglandular resection and liposuction.

In this paper we present the first ever reported case of Mondor’s disease in male patient after surgical correction of gynecomastia with liposuction assisted skin sparing adenectomy.

## Case report

**Figure 1.**
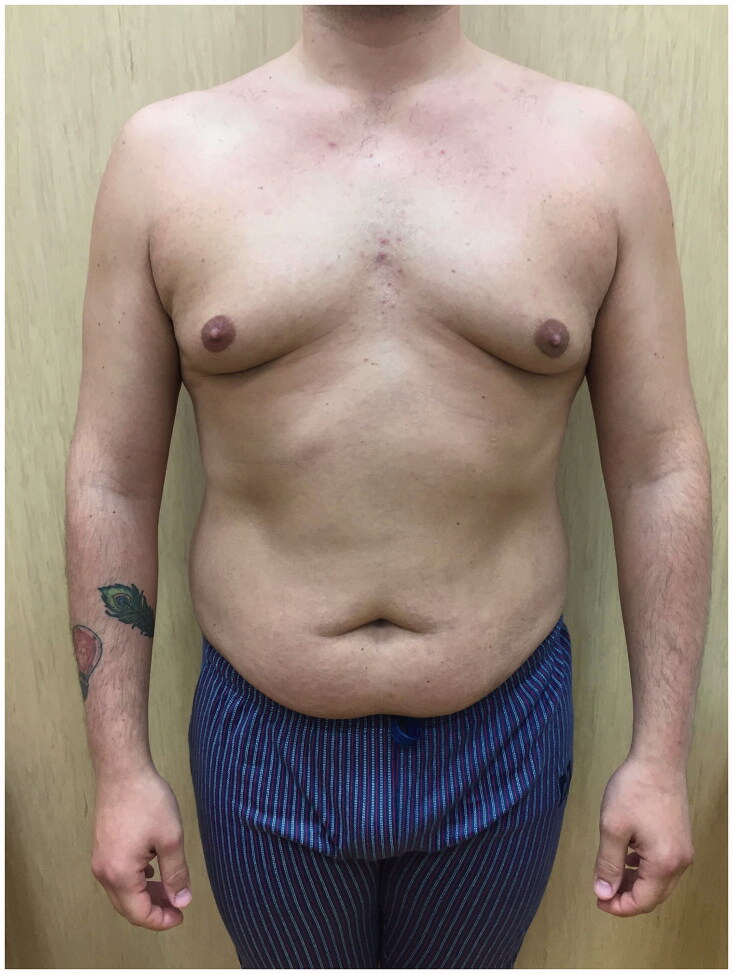
Preoperative photo showing grade II gynecomastia.

A 26 years-old Caucasian healthy male with grade II gynecomastia underwent liposuction assisted skin sparing adenectomy with inferior periareolar incision (Figure 1).

Suction drains were bilaterally positioned and left in place for 48 h postoperatively and elastic thoracic compressive vest was placed at the end of the procedure and continuously worn by the patient for one month after the surgery.

The postoperative course was uneventful and stitch removal occurred at 15^th^ postoperative day.

**Figure 2.**
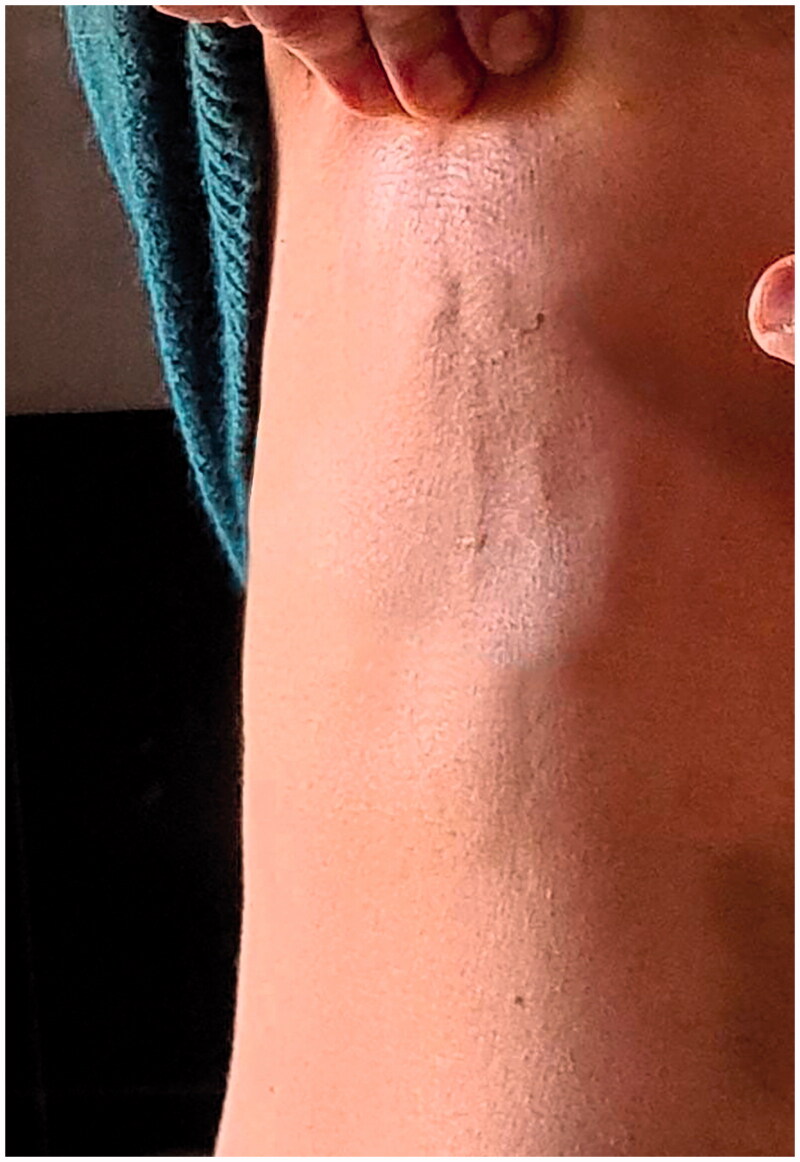
Mondor’s Disease of the right breast.

Five weeks after the surgery and one week after the removal of the compressive vest the patient noted the appearance of two linear cord-like subcutaneous lesion extending vertical from the lower aspect of the right areola to the abdomen (Figure 2).

Although these lesions did not cause any symptoms, the patient became very frightened and sent us a photo.

We advised the patient to start topical NSAID and to swab for COVID-19, which came back negative.

The next day he came to our unit for a check-up and the cord-like lesions were no longer visible but only palpable and mildly painful when palpated.

No other symptoms were reported and the patient denied personal and familiar history of thromboembolic diseases.

Hemogram, ESR, autoantibody, liver function and coagulation labs were obtained, demonstrating results within normal laboratory value range.

**Figure 3.**
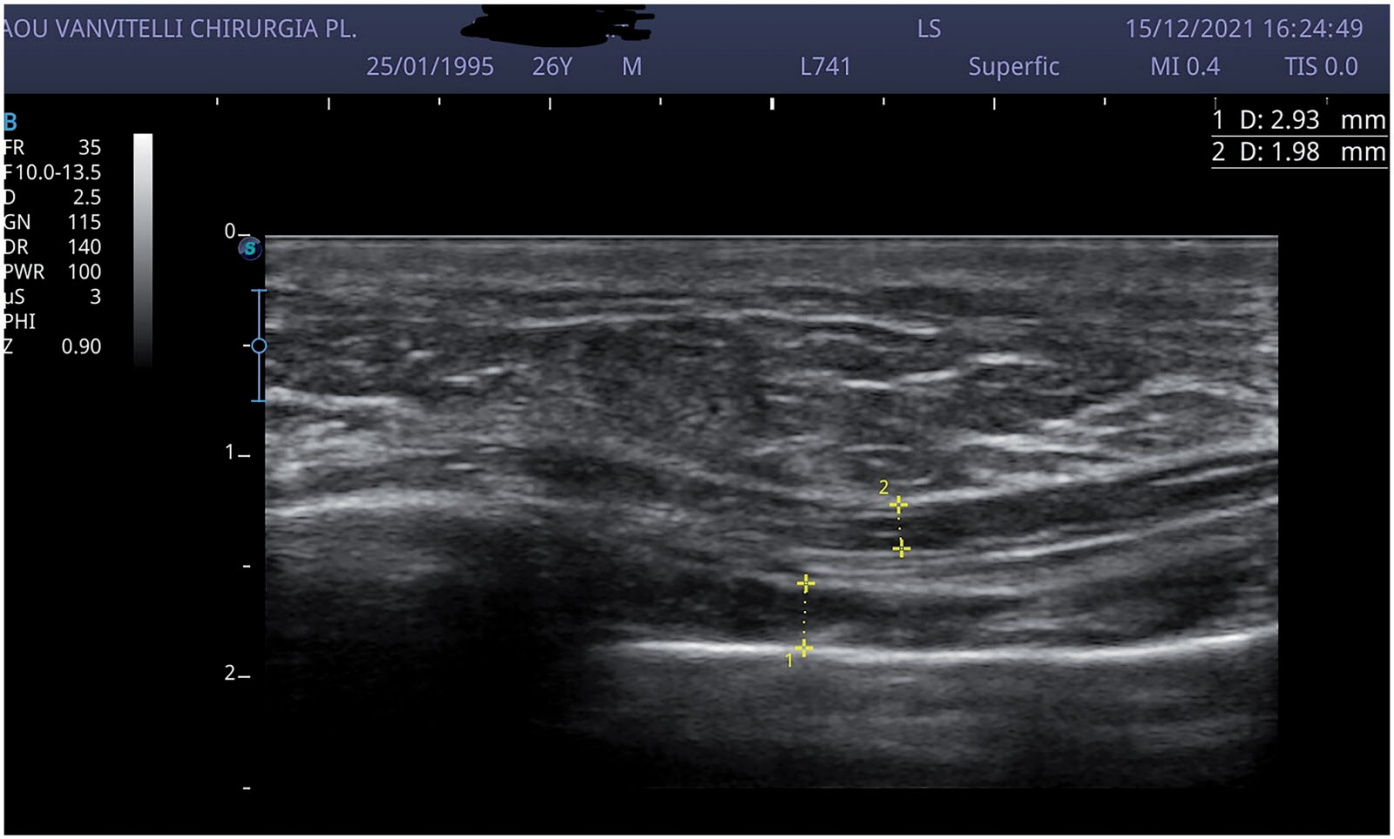
US scan showing thrombophlebitis of the right thoracoepigastric vein.

Breast and upper abdomen US scan demonstrated the presence of thrombophlebitis of the right thoracoepigastric vein with no sign of fluid collection in the breast (Figure 3).

The patient started subcutaneous administration of Low Molecular Weight Heparin (LMWH) and topical NSAID for three weeks.

**Figure 4.**
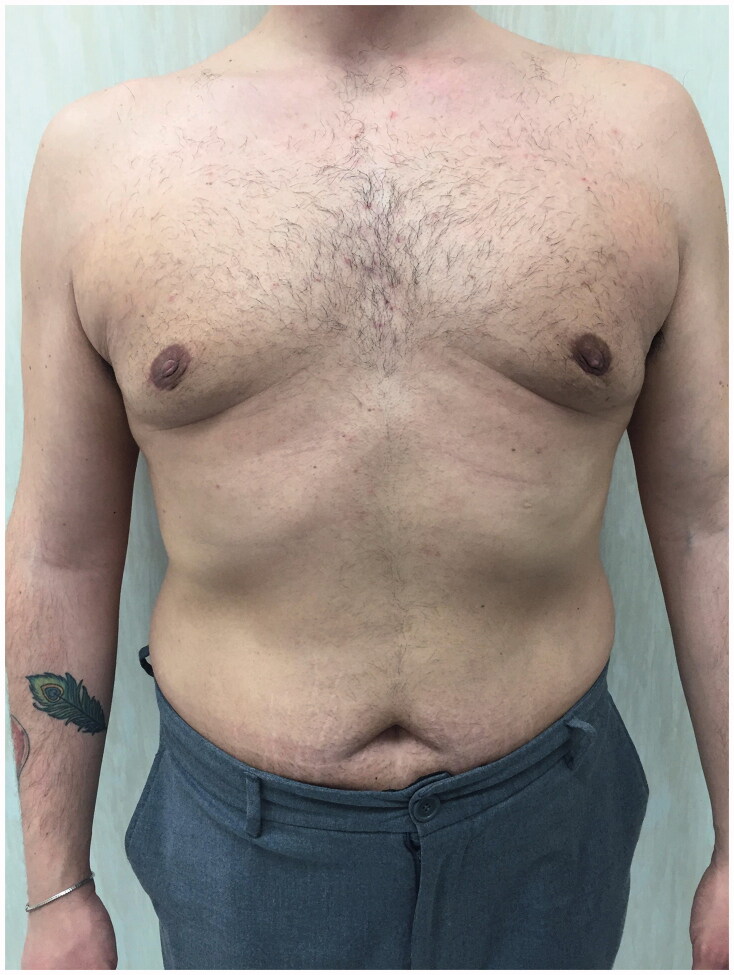
Postoperative photo at 2 months after surgery.

A good response to the established treatment was registered in five days and in two weeks the thrombophlebitis disappeared (Figure 4).

## Discussion

Mondor’s disease is a rare form of superficial thrombophlebitis that can be either idiopathic, iatrogenic or traumatic.

Traumatic MD of the breast in female patients has been related to excessive physical activity and a tight bra.

Idiopathic MD has been described as a paraneoplastic syndrome in breast cancer [8–10] and in cutaneous metastasis [11–13] of any type of cancer. [14]

Iatrogenic MD of the breast has been always described in female patients as a complication in every breast procedures both surgical [15–18] and diagnostic [19].

In males MD is described exclusively at the penile level and is defined as Penile Mondor’s Disease (PMD).

The etiology of PMD is essentially related to Virchow’s triad.

In fact vessel-wall can be damaged by intense sexual activity, a prolonged erection can lead to blood stasis and hypercoagulation can be related to infections, trauma, surgery or occult tumors of the genital sphere [4,5,20–23].

Clinical signs of MD are cord-like palpable indurations composed of occluded superficial mainly located at level of the anterolateral thoracic wall or at level of the inframammary fold.

For what concerns PMD, indurations appear on the dorsum and the dorsolateral aspect of the penis as a consequence of the occlusion of superficial veins of the lower abdominal wall.

Some patients affected by MD and PMD suffer pain, fever, erythema and discomfort during physical activity.

A standard management for MD has not been established yet because it is an uncommon clinical entity and tends to spontaneous resolution within one or two months without any therapy.

In painful cases administration of NSAID has been demonstrated to be effective in pain relief [24].

Administration of subcutaneous low-molecular-weight-heparin (LMWH) has been reported as effective in counteracting the blood hypercoagulability in the acute phase of the MD [25,26].

Regarding idiopathic MD it is crucial to determine the underlying cause of the thrombophlebitis and treat it with higher priority, because MD alone is not a life threatening condition.

Gynecomastia is defined as a feminizing deformity of the male thorax characterized by hypertrophic breast tissue with multifactorial etiology.

In 2003 Rohrich [27] classified this clinical condition into four grades, according to the severity and structural composition of the breast tissue.

This simple and rigorous classification allows surgeons to choose different surgical approaches based on the clinical situation they are facing.

Many surgical techniques have been described for treatment of gynecomastia such as skin sparing adenectomy, periareolar reduction adenectomy, reduction mammaplasty with dermoglandular resection and liposuction. This latter option can be used alone or in combination with other ablative procedures [28].

The most frequent complications in the surgical treatment of gynecomastia are hematomas and seromas, for this reason suction drains are placed and a compression chest garment is strictly recommended for at least one month after surgery [29,30].

Recovery and rehabilitation following the surgical correction of gynecomastia are the same for breast surgery in women [31,32].

In this paper we reported the first case of Mondor’s disease as a complication of a liposuction assisted skin sparing adenectomy for gynecomastia, which is also the first MD described in the male breast.

In conclusion we believe that the fact that MD has been widely described in female breast surgery, combined with the rarity and self-limiting nature of the disease itself, has led to the underestimation of this complication in male breast surgery.

Our hope is that this article will be a thoughtful starting point for including MD in the informed consent of all breast surgical procedures, regardless of the patient’s gender.
